# Serum Folate and Methylenetetrahydrofolate Reductase (MTHFR) C677T Polymorphism Adjusted for Folate Intake

**DOI:** 10.2188/jea.JE2007417

**Published:** 2008-05-29

**Authors:** Kazuko Nishio, Yasuyuki Goto, Takaaki Kondo, Shimon Ito, Yoshiko Ishida, Sayo Kawai, Mariko Naito, Kenji Wakai, Nobuyuki Hamajima

**Affiliations:** 1 Department of Preventive Medicine/Biostatistics and Medical Decision Making, Nagoya University Graduate School of Medicine; 2 Research Fellow of the Japan Society for the Promotion of Science DC; 3 Department of Medical Technology, Nagoya University School of Health Sciences

**Keywords:** Eating, Folic Acid, *MTHFR* C677T, Japanese

## Abstract

**Background:**

Serum folate concentration is lower in individuals with the *methylenetetrahydrofolate reductase* (*MTHFR*) 677TT genotype than in those with the *MTHFR* 677CC or 677CT genotypes. Since studies considering folate intake are limited, we examined the association between folate intake and serum folate levels, according to the genotype.

**Methods:**

The subjects comprised 170 Japanese persons (74 males and 96 females) aged 20-75 years who visited a clinic to test for *Helicobacter pylori* infection. Folate intake was estimated using a semiquantitative food-frequency questionnaire, and serum folate was measured in the residual fasting blood samples of the subjects. *MTHFR* C677T was genotyped using polymerase chain reaction.

**Results:**

The geometric means of serum folate level were 6.19, 6.20, and 5.17 ng/mL among the 60 participants with the 677CC genotype, 90 participants with the 677CT genotype, and 20 participants with the 677TT genotype, respectively. No difference was noted in the mean folate intake estimated using the food-frequency questionnaire. Regression analysis showed that log_e_(serum folate) adjusted for age, sex, and log_e_(folate intake) was significantly lower among those with the 677TT genotype than among those with the 677CT or 677CC genotypes (p = 0.01). The adjusted reduction in serum folate was 20.2% (95% confidence interval, 5.4-32.6%) in the case of the 677TT genotype relative to the levels in the case of the 677CC/677CT genotypes. When folate intake was adjusted for total energy intake, using the residual method, the slope of the regression line for 677TT was smaller than those of the regression lines for 677CC and 677CT.

**Conclusion:**

Individuals with the 677TT genotype may need to consume more folate to maintain serum folate levels similar to those found in individuals with the 677CC/677CT genotypes.

## INTRODUCTION

Shortage of dietary folate has been proven to elevate homocysteine levels.^1^^-^^3^ Folate supply substantially reduces the plasma homocysteine levels among individuals with hypercysteinemia. Low serum folate and the resultant high plasma homocysteine levels are one of the causes of neural tube defects (NTDs) in children;^4^^,^^5^ neuropsychiatric conditions;^6^ cardiovascular diseases, including atherosclerosis;^7^^-^^10^ and cancers.^11^ For the prevention of NTDs, official bodies worldwide recommend that women take 400 µg folate/day before conception and during early pregnancy. However, the implementation of this recommendation is difficult. Although folic acid supplements are effective in optimizing the folate status in women,^12^ they are not an effective strategy for the primary prevention of NTDs due to poor patient compliance.^13^ Therefore, mandatory fortification of grain products with folic acid was introduced in several countries, including the United States^14^ and Canada.^15^ Despite an impressive decrease in the prevalence of NTDs after this fortification,^16^^,^^17^ the policy remains controversial. The fortification not only delivers the required nutrient levels to the high-risk group but also provides high-dose folate products to a proportion of the general population. Of the greatest concern is the potential for the high intake of folic acid that in turn masks anemia resulting from vitamin B_12_ deficiency in the elderly, thereby allowing the concomitant irreversible nerve degeneration to go undetected.^18^ Moreover, the United States and Canada have experienced abrupt reversals of the downward trend in colorectal cancer incidence concurrently with the nationwide fortification of enriched uncooked cereal grains with folic acid.^19^ A recent large intervention study showed that 1 mg/day folic acid might increase the risk of advanced lesions and adenoma multiplicity.^20^

In the metabolic pathway involved in the conversion of folate to homocysteine, 10-methylenetetrahydrofolate reductase (MTHFR, EC 1.5.1.20) is one of the key enzymes, others being methionine synthase and cystathionine beta synthase. MTHFR metabolizes 5,10-methylenetetrahydrofolate (THF) to 5-methyl-THF-the primary circulating form of folate. In the gene that codes MTHFR, a C to T polymorphism was reported at position 677 (*MTHFR* C677T), which causes the substitution of alanine with valine. This substitution leads to a 30% decrease in the enzyme activity in heterozygotes and a 60% decrease in homozygotes.^21^ The frequency of the T allele approaches 30% in many ethnic groups.^22^ Several studies have shown that hyperhomocysteinemia was more frequent among individuals with the TT genotype than among those with the CC genotype.^1^^,^^7^^,^^23^

Genotype has also been associated with plasma or serum folate concentrations.^24^^-^^31^ However, studies regarding dietary folate intake are relatively rare. Therefore, this study aimed to assess the effect of the *MTHFR* C677T genotype with adjustments for folate intake. We also examined the association between folate intake and serum folate levels, according to the *MTHFR* C677T genotype.

## METHODS

### Study Subjects

The subjects were individuals who visited the Daiko Medical Center, Nagoya University in Nagoya, Japan, and provided written consent to participate in the present study. The Center provided preventive care that was not covered by health insurance. From July 20, 2004 through December 1, 2005, 220 individuals visited the Center to undergo *Helicobacter pylori* infection tests and subsequent eradication treatment. Those with gastric cancer (n = 8), idiopathic thrombocytopenic purpura (n = 3), or chronic urticaria (n = 5) were excluded from the present study. Among the remaining 204 visitors, 1 person who was 19 years old and 19 persons who did not provide consent were also excluded. Blood for research purposes could not be drawn from 9 persons. The quantity of 4 of the blood samples was insufficient for the measurement of serum folate, and the semiquantitative food-frequency questionnaire (FFQ) was not administered to 1 participant. Analyses were conducted for the remaining 170 participants.

This study was approved by the Nagoya University School of Medicine Ethics Committee (approval No.155, issued on June 2004).

### Lifestyle Questionnaire and Biomarker Measurements

Participants were asked to complete a self-administered questionnaire with questions regarding the following: sex, age, supplement use, drug use, alcohol use, smoking, and disease history. Participants who had smoked less than 100 cigarettes in their lifetime were categorized as never smokers, and the rest were categorized as ever smokers. Individuals were categorized as current alcohol drinkers if they drank alcoholic beverages at least once a week and as non-drinkers otherwise. The participants were also asked to complete a validated FFQ developed by Takahashi et al.^32^ The reproducibility and validity of this FFQ have been measured for most nutrients but not for folate.

The study participants were required to fast overnight for a urea breath test for identifying *H. pylori*. In the morning, peripheral blood was drawn for anti-*H. pylori* antibody tests. The blood samples were treated in an indoor laboratory. All serum samples were obtained by centrifugation within 3 h of blood collection, with a few exceptions (maximum 5 h). Hemolysis was not visually observed in any serum sample. The residual sera of the participants were preserved at -40°C and used for serum folate measurement. The serum folate levels were measured using the chemiluminescence enzyme immunoassay method. The laboratory staff was blinded to the genotype of the subjects.

### Genotyping

DNA was extracted from the buffy coat and preserved at -40°C, using BioRobot^®^ EZ1 (Qiagen Group, Tokyo). The *MTHFR* C677T polymorphism was genotyped using polymerase chain reaction (PCR) with confronting two-pair primers.^33^ Each 25-µL reaction tube contained 50-80 ng DNA, 0.12 mM dNTP, 12.5 pmol of each primer, 0.5 U AmpliTaq Gold (Perkin-Elmer, Foster City, CA), and 2.5 µL 10× PCR buffer, including 15 mM MgCl_2_. PCR was conducted with initial denaturation at 95°C for 10 min, 30 cycles of denaturation at 95°C for 1 min, annealing at 60°C for 1 min, extension at 72°C for 1 min, and a final extension at 72°C for 5 min. The primers were F1: 5′-AGC CTC TCC TGA CTG TCA TCC-3′, R1: 5′-TGC GTG ATG ATG AAA TCG G-3′, F2: 5′-GAG AAG GTG TCT GCG GGA GT-3′, and R2: 5′-CAT GTC GGT GCA TGC CTT-3′. The length of the amplified DNA fragments was 128 bp for the C allele, 93 bp for the T allele, and 183 bp for the common band.

### Statistical Analysis

The distributions of serum folate levels and dietary folate intake were skewed; therefore, natural logarithmic transformation was applied for data analyses. Energy-adjusted dietary folate intake was also estimated using the residual method.^34^ Log-transformed serum folate was examined using analysis of covariance and linear regression analysis. Hardy-Weinberg equilibrium was applied to test for genotype frequency. All statistics were calculated using the computer program STATA^®^ Version 8.2 (StataCorp LP, College Station, TX). The effect of the interaction between genotype and dietary folate intake on serum folate levels was assessed using a multivariate linear regression model. In this assessment, the log-transformed continuous energy-adjusted residuals of dietary folate intake were used. The p-value for the interaction was calculated using the interaction term of the genotype group multiplied by the continuous log (dietary folate intake). Two-sided *P*-values less than 0.05 were considered to be statistically significant. Adjustments for multiple comparisons were not performed in any analyses because this was an exploratory study.

## RESULTS

A total of 170 Japanese subjects (74 males and 96 females) aged 20-73 years were included in this study. The frequency of the *MTHFR* C677T polymorphism among subjects with the CC, CT, and TT genotypes was 35.3%, 52.9%, and 11.8%, respectively. In this study, the *MTHFR* C677T genotype frequency did not deviate from the Hardy-Weinberg equilibrium (*P* = 0.12).

The characteristics of patients, according to the *MTHFR* C677T genotype, are shown in Table 1. No difference was present in the geometric mean of folate intake, as estimated using the FFQ: 252.4, 243.8, and 263.0 µg/day in the case of the CC, CT, and TT genotypes, respectively. No significant differences were observed in the age, sex, serum folate, dietary folate intake, dietary vitamin B_6_ intake, dietary vitamin B_12_ intake, energy intake, supplement use, smoking status, and alcohol consumption status.

**Table 1.** Characteristics according to *methylenetetrahydrofolate reductase* (*MTHFR*) C677T genotype.

Characteristics	*MTHFR*			P^*^

	CC (n = 60)	CT (n = 90)	TT (n = 20)	
	(Mean ± standard deviation)			
Age (years)	51.1 ± 11.1	50.7 ± 11.9	47.9 ± 14.5	0.57
Vitamin B_6_ intake (mg/day)	6.8 ± 3.0	6.9 ± 3.0	7.5 ± 3.2	0.55
Vitamin B_12_ intake (µg/day)	265.3 ± 79.2	256.0 ± 78.9	278.7 ± 97.7	0.67
Energy intake (kcal/day)	1804.3 ± 341.4	1805.1 ± 366.0	1836.3 ± 492.7	0.94
Folate intake (µg/day)	264.3 ± 79.2	256.0 ± 78.9	278.7 ± 97.7	0.51

	(%)			
Sex (% of males)	40.0	47.8	35.0	0.46
Vitamin B complex or folic acid supplement users	5.0	3.3	5.0	0.55
Ever smokers	5.0	14.4	15.0	0.17
Current alcohol drinkers	71.7	75.6	65.0	0.72

* : Analysis of variance (ANOVA) was used to test the difference in continuous variables, and the chi-square test was used for categorical variables.

The geometric mean of the serum folate level was 6.19 ng/mL among the 60 participants with the 677CC genotype, 6.20 ng/mL among the 90 participants with the 677CT genotype, and 5.17 ng/mL among the 20 participants with the 677TT genotype. Table 2 shows the sex- and age-adjusted means and the standard error of the log-converted serum folate levels according to the *MTHFR* C677T genotype. No significant difference was detected in the mean serum folate level among the different genotypes. However, among the subjects with a high dietary folate intake (i.e., 50% of all the subjects studied), age-, sex-, and energy-adjusted regression analyses revealed that the log-converted mean was significantly lower in subjects with the TT genotype than in those with the CC genotype (*P* = 0.01). In contrast, the difference was not significant in the remaining subjects, who had a low dietary folate intake.

**Table 2.** Age- and sex-adjusted mean and standard error of logarithmic-transformed serum folate level (ng/mL).

		Sex- and age-adjusted^*^			Sex-, age-, and energy-adjusted^†^		

		CC	CT	TT	CC	CT	TT
All subjects	log(serum folate)	1.81 ± 0.05	1.83 ± 0.04	1.64 ± 0.09	1.81 ± 0.05	1.84 ± 0.04	1.63 ± 0.09
(n = 170)	n	60	90	20	60	90	20

Dietary folate intake							
Low (<255 µg/day)	log(serum folate)	1.65 ± 0.07	1.73 ± 0.06	1.49 ± 0.12	1.67 ± 0.07	1.72 ± 0.06	1.63 ± 0.12
(n = 85)	n	28	47	9	28	47	9
High (>255 µg/day)	log(serum folate)	1.97 ± 0.07	1.94 ± 0.06	1.77 ± 0.13	1.94 ± 0.07	1.96 ± 0.06	1.60 ± 0.11^‡^
(n = 85)	n	32	43	11	32	43	11

* : Adjusted for age and sex by using analysis of covariance (ANCOVA)

† : Adjusted for age, sex, and energy intake by ANCOVA

‡ : Siginificantly different from CC genotype: *P*=0.01

**Figure 1.**
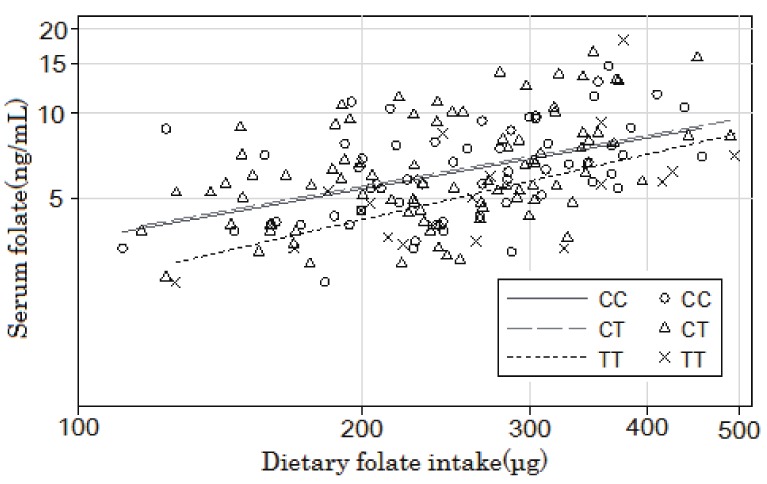
Regression lines for each *MTHFR* genotype on a logarithmic scale obtained by plotting the serum folate concentration against the dietary folate intake.

Figure 1 shows the unadjusted regression lines for each *MTHFR* genotype on a logarithmic scale obtained by plotting the serum folate concentration against the dietary folate intake (non-energy adjusted values). The adjusted analysis for age and sex showed a significant positive relationship between the logarithm of serum folate (y) and that of dietary folate intake (x) in the CC and CT groups (CC: y = 0.56x - 1.38, *R^2^* = 0.29, *P* for slope = 0.001 and CT: y = 0.44x - 0.87, *R^2^* = 0.29, *P* for slope = 0.003), whereas the corresponding relationship in the TT group was marginally significant (y = 0.65x - 2.00, *R^2^* = 0.54, *P* for slope = 0.07). Regression analysis showed that the logarithm of serum folate adjusted for age, sex, and logarithm of folate intake was significantly lower among subjects with the 677TT genotype than among those with the 677CT or 677CC genotypes. The reduction in the serum folate level was 20.2% (95% confidence interval: 5.4-32.6%) for the 677TT genotype relative to those for the 677CC/677CT genotypes.

**Figure 2.**
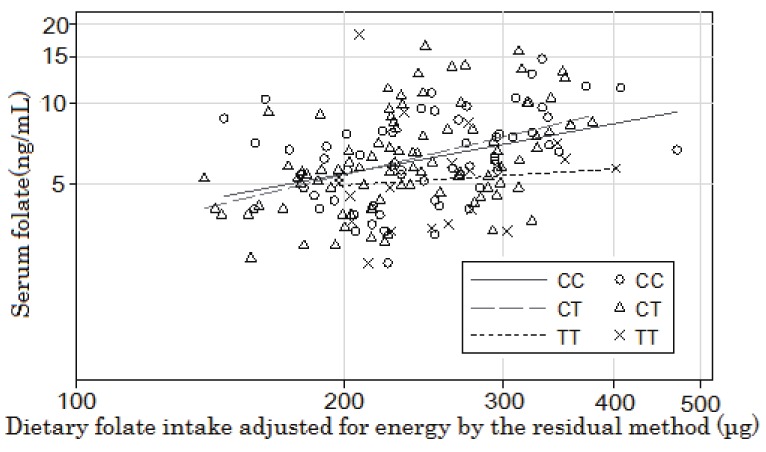
Relationship between the *MTHFR* genotype and the serum folate level and the effect of energy-adjusted dietary folate intake on the latter.

Figure 2 shows the unadjusted regression lines obtained by plotting the logarithmic serum folate levels against the log_e_(dietary folate intake) values that were energy-adjusted by the residual method for each genotype group. The age- and sex-adjusted slope was significant in both the CC and CT genotype groups (CC: y = 0.55x - 1.91, *R^2^* = 0.29, *P* for slope = 0.007 and CT: y = 0.59x - 2.00, *R^2^* = 0.31, *P* for slope = 0.002). The corresponding slope in the TT group was small and not significant (y = 0.09x + 0.80, *R^2^* = 0.83, *P* for slope = 0.78). However, the slopes of the regression lines adjusted for sex and age did not significantly differ between the CC + CT and TT genotypes (*P* = 0.13 for the interaction).

When 7 supplement users were excluded, no substantial changes were seen in these results (data not shown); 6 of them had taken vitamin B supplements not containing folic acid, and 1 had taken folic acid supplements. Similarly, adjustment for alcohol consumption did not substantially change the results (data not shown).

## DISCUSSION

This cross-sectional study aimed to evaluate the effect of the *MTHFR* C677T polymorphism on the association between serum folate levels and dietary folate intake. It has been reported that subjects with the TT genotype of *MTHFR* C677T have lower serum folate levels; however, it is necessary to compare the levels under conditions of equal dietary folate intake. Our results confirmed that subjects with the TT genotype had lower serum folate levels even after adjustments for the dietary folate intake. The analysis without energy-intake adjustment showed that serum folate levels were lower among subjects with the TT genotype than the levels among subjects with other genotypes; the average reduction was approximately 20%. On the other hand, sex-, age-, and energy-adjusted regression analyses showed that remarkably lower serum folate levels were present in subjects with the TT genotype who had a high folate intake. However, this did not explain which estimates were correct, i.e., the ones in the subjects with high folate intake or in those in the subjects with low folate intake. Previously, 3 studies have reported the association between serum folate and *MTHFR* C677T while taking dietary folate intake into account; the results were inconsistent. One study reported no difference in the serum folate levels among subjects with different *MTHFR* genotypes.^35^ In another study, a remarkable reduction in the serum folate concentration was reported among subjects with the TT genotype and low dietary folate intake.^36^ The third study reported a significant reduction in serum folate levels among subjects with this genotype who had high dietary folate intake.^37^ The latter 2 studies estimated dietary folate intake using a validated FFQ, in which energy intake was not adjusted.

In general, nutrient intakes show a positive correlation with the total energy intake. This tendency is particularly strong in the case of the estimates obtained using FFQs. Therefore, when the association between nutrient intake and disease is analyzed, the influence of the total energy intake should be excluded. In the simplest method involving the calculation of nutrient density, the crude nutrient intake is divided by the total energy intake; however, this does not completely remove the influence of total energy intake. Therefore, the method of residuals is often used during linear regression analysis. The residual method appears to be more effective for detecting associations with disease risks. Generally, the residual and nutrient density methods yield similar results.^34^ The body size of the participants was not adjusted in this analysis. Although there were no extreme observations, an analysis adjusted for energy intake could partly remove the influence of the difference in body size.^38^ However, this may not be applicable to the identification of dose-response relationships according to the *MTHFR* genotype.

We determined the *MTHFR* genotype of 170 individuals and found that 35.3% were homozygous CC, 52.9% were heterozygous CT, and 11.8% were homozygous TT. These frequencies were similar to those reported in other Japanese subjects.^39^^-^^41^ Although the present subjects consisted of visitors to an *H. pylori* clinic, the results may be extended to the general population in Japan.

Our results suggested that subjects with the TT genotype had approximately 20% lower serum folate levels, based on the sex- and age-adjusted analyses. This indicates that people with the TT genotype may have to consume more dietary folate than people with the CC or CT genotypes. Because the slope in the case of subjects with the TT genotype in the sex- and age-adjusted analyses was 0.65, approximately 1.4 (exp[(log{1/ (1 - 0.2)}/0.65]) times higher intake may be required to ensure that these individuals have the same level of serum folate as those in individuals with other genotypes.

The bioavailability of food folates relative to folic acid supplementation ranges between 10% and 98%, depending primarily on the method of fortification.^42^^-^^47^ The uncertainty regarding folate bioavailability^48^ is of particular concern in countries where folic acid fortification is not adopted or permitted^45^ because such countries depend largely on natural food folates to optimize nutritional status. In the United States, folic acid fortification is mandatory and relatively less reliance is placed on natural folate sources. Thus, the dietary recommendations are based mainly on the bioavailability of folic acid added to food, and less on natural food folates, resulting in the introduction of dietary folate equivalents (DFEs).^49^ The estimated DFE conversion factor of 1.7 is primarily based on a metabolic study in non-pregnant women that estimated the bioavailability of food folates as no more than 50% that of folic acid.^45^ Another study based on folic acid-fortified food showed that the bioavailability of free folic acid was 85%.^50^ Very few people use folic acid supplements in Japan. The largest source of dietary folate is vegetables (38-58% of total intake).^51^ Moreover, dietary folate is different from folic acid, in which the risk of the excess intake is not reported. Therefore, the estimated average requirement of folate may have to be calculated by focusing on individuals with the TT genotype. We must further explore the methods of optimizing folate intake in individuals with the TT genotype.

Our study had several limitations. First, this study had a relatively small sample size because it was an exploratory analysis. The results of subgroup analysis, such as the coefficient for the TT genotype in the non-energy adjusted analysis, were not significant because of the low statistical power (48.7% for a two-sided test with alpha error = 0.05 when the slope for the TT genotype in the population was assumed to be 0.65 and its standard deviation = 0.34). However, the sample size was sufficient for the main purpose, i.e., demonstrating significant differences in the serum folate concentrations among individuals with different *MTHFR* genotypes after adjusting the folate intake. Second, the reproducibility and validity of the FFQ used in our study have not been reported for folate, although the FFQ is reasonably reproducible and valid for many other nutrients. The correlation coefficients for the intakes of the major sources of folate determined using the FFQs and weighed dietary records for 7 continuous days were moderate: 0.462 for green-yellow vegetables and 0.635 for fruits.^32^

In conclusion, this study confirmed that Japanese people with the TT genotype had lower serum folate levels than those in people with the CT or CC genotypes, even after the folate intake was adjusted. The slope of the regression line depended on the method used to estimate folate intake, particularly, in the case of people with the TT genotype. These results highlight the need for individuals with the TT genotype to consume slightly more than the recommended folate dose through natural foods or folic acid supplements.
